# Supplementary material for: Patterns of Intron Gain and Loss in Fungi
Source: PLoS Biol. 2004 Nov 30;2(12):e422. doi: 10.1371/journal.pbio.0020422 (PMC532390; doi:10.1371/journal.pbio.0020422)
Supplement: Table S1 — Also available at http://genes.mit.edu/NielsenEtAl/. (4.3 MB ZIP). [file pbio.0020422.st001.zip › NielsenEtAl/html/815.html]

AN0037.1.NCU00273.1.MG08911.1.FG05518.1


```
 CLUSTAL W (1.82) Multiple Sequence Alignments - Introns Inserted


Sequence 1: MG08911.1	1666 aa
Sequence 2: FG05518.1	1642 aa
Sequence 3: NCU00273.1	1730 aa
Sequence 4: AN0037.1	2619 aa
Alignment Length: 2717 aa
Number Identitical Residues: 359 aa
Alignment Score (without introns) 24598


MG08911.1 	MSEAYLLETLQSFYGDLIAIGEGRLNGPSLEGDELTALLEN--FKNFLEEPPKRDASRKQ
NCU00273.1	MAELRKFESLYALHTELQAVSQQRYEELQTVEQLLEQHADS--FSKFLDKPPRNPTSRTA
FG05518.1 	MAEITALDALQAFHQGLLSLREGRVEGAETLGNEFLVQVFETELAKFWDKPARKSDNRNA
AN0037.1  	MESAEPLEGLRGLYQDLSALSDSSLLNIDRLRVELETHIHD--FRTLLDKPTKSNESRMS
          	* .   :: * .::  * :: :      .     :     .  : .: ::*.:   .*  

MG08911.1 	LESG1KISVGDVEYSINKEFQEYSIQLADEVNLDEVEAAKLLLEAQD--SQILLGRSLVE
NCU00273.1	LQSG~RIKVQDEEYSVTQSFINDALKLADELDLDEVEAARVLLDADAEGDQASFDRPLWV
FG05518.1 	VKSG1KVVIDGDEYSINDNFQQDVLTFSDEVELDELESTRCLLESQE--DPNTLGRSLLE
AN0037.1  	VLSG1KITIDDVEYSVNDEFQQGTLQVADALDLDELLAALLFFKAQET--SQEYDRPPVI
          	: ** :: : . ***:...* :  : .:* ::***: ::  ::.::        .*.   

MG08911.1 	CALIRFHQRRKYLLDCIRLCIELAND-DENESIKAVFEEIVARYVFDLPLPGAPAAAVPK
NCU00273.1	CGLLRFQNERSYLLDCMRLCIQIANDEDIDPSVQEGFGQVVDERIFGIPAQGS--KPQAG
FG05518.1 	CAIIRFHQQRKYVLDIVRLLLELEGL-DDD-------------------------IADAE
AN0037.1  	TAIINFHQRRHFLLESLRLIFQESFEVEREATQEMMQEMLAHVVEIK-------DGQLRN
          	 .::.*::.* ::*: :** ::     : : : .                          

MG08911.1 	EKKVVPR~CMAAMAKIKDWLEKLANKIMLARMHSGTAP----PEIETIEFCRLSLVQQHE
NCU00273.1	AQKFVPK~CVAGLQAIRSTLQSIGEKVAAQNVLFQPNVSKRMDQQEPSEIMRLKLIEQHE
FG05518.1 	ALESVKL2CMASMREIKAWLQKIGDKIAAAQTLGQTTTNGVSEQIETIEFSRVSLMQQHE
AN0037.1  	ASLYTRK~CLKSMEDIEKWLSLLGEQIQKASIVGQSEDQ---LVMEAIEHQRFTLLQQHE
          	    .   *: .:  *.  *. :.:::        .  .      *. *  *..*::***

MG08911.1 	NLAVILCAAVEQRHAERTNFEEFIQLLKKADKYDHLLV1HLFPVIGAYIRLYGSTEGWGD
NCU00273.1	TLSLILCAAVEKKQAESKDFKEFIQLLRKVDKYDHLLV1HLIPVLGAYIATFGSNVGGGD
FG05518.1 	LLGVILCRSIEMRQGSTQDFLDFVSVLQKVDRYDALLV~HLVPSIGAYISVFGSAEGGYD
AN0037.1  	SLGAILCYLFKGPYTSPEDLRVLLKHLRKLDRFDGVLV~HYVPAIIASFVQHGSPERSGN
          	 *. ***  .:    .  ::  ::. *:* *::* :** * .* : * :  .**     :

MG08911.1 	LVIARSLNQKIVN-DETS-WSLPFLYAAVKVWWIAEYSGFYVEGP---VSDPSIDIDKED
NCU00273.1	VEPARQLNEFICGRTAEDVWAIPSLGAAVRAWWIAEYSGWYHDDYAG-YEARGINLDKED
FG05518.1 	LIKARELHGKLFP-TDSSPWPLSQLQAAFRAWWLAEYSGFYVDDPPEAAIPPNTDLDEED
AN0037.1  	YQEARSLNTAVISTKDGQNWAHQPFHSAVIALWLSVYSAWDYDGPSS--APPGVDLEKEA
          	   **.*:  :      . *.   : :*. . *:: **.:  :. .      . ::::* 

MG08911.1 	KERNKQFTDALKEGAFDFMLSLAGDVAAP--EWQDPSRIGIRQWLQRKSPQLMT------
NCU00273.1	EERTKQFMDALKDGAFDYLLAVAADCHAHNQDWQDPTRWGMRQWLQRKTTPLAS------
FG05518.1 	RQRSKAFLESLKDGSFDFLLSIAADVKSP--DWHDPVRAGMRKWLQRKSPALAP------
AN0037.1  	EERTKTFMTALDDGGLDLLVSICSGVSGE--EWSDPARSELVTMLLRESASWLKESGSAT
          	.:*.* *  :*.:*.:* ::::...  .   :* ** *  :   * *::.    .:.:::

MG08911.1 	-DSTPFSDYFHTCLMNQLEDFVDAFISNLPDVLRKLRTEEDEQRQLSQHHEQD-------
NCU00273.1	-EALPFAPHFHSCLMTHLEVLIDAAISNIPDVLRKLRTEEDEQRQLSQTHEQD-------
FG05518.1 	-DAIPFADFFQMSLMLQLEVFVDGFISNLPDVLRKLRVEEDEQRQSSQTHEQD-------
AN0037.1  	LEADSCSPYLKTLLMENFEVFAESCIANMPDAVRRLKTEEDSQRLDQITALRDGLTSNLH
          	 :: . : .::  ** ::* : :. *:*:**.:*:*:.***.**  .    :*. ::.  

MG08911.1 	-------LDLERFLVIISYVFERRPDAADVFWSDSESNLAGFMQWASRRASTPLVCAFCE
NCU00273.1	-------LDLERFLMIIAYAYDGRPDAAMTFWEDTESNLAGFLNWASRRASTPLVSAFCE
FG05518.1 	-------LDLERFLLIIAYAYEGRPDAAMNFWSDPDSNLAGFMHWASRRASTPLVTAFCE
AN0037.1  	RDLVEARTHLESFLLIMAFAFEGRPDAAQEFWADPDGNLYGFLQWSSKRQTVPRVSAFCE
          	 .  .:  .** **:*:::.:: *****  ** *.:.** **::*:*:* :.* * ****

MG08911.1 	MLQSITENEQYALAAHQFLLED-SNAGGKMRRSQSLTWAQIFKELHYFTKKIRGEISSPQ
NCU00273.1	MLQALAANEECATAAHNFLLDETTHSGGKLKRSASLTWNQIFKELKFFMEKLQTKPLPAP
FG05518.1 	MLQAISENEECASAAHEFLIDEGHHSSGKMRKSQSLTWAQIFKELDFFSAKIR-QKPAPP
AN0037.1  	LLCSISGGEDNAAAAHRFLLEEDKFMSSKFKRSASMNWAQMFAELQIYATRITEKPSTAQ
          	:* ::: .*: * ***.**:::    ..*:::* *:.* *:* **. :  ::  :  .. 

MG08911.1 	QIHEYRTQKPGEDLAETEPESAMLLECYLRLIARLGSQSTAAREFLLRNQDFPLVDVILQ
NCU00273.1	SQLVRHHAKPSSDQAETEPESAMMLECYLRLIAKLGYESPVARNLLLLDTSVELPETLIR
FG05518.1 	QAIRYRNEKMSSDQAETEPESAMMLECYLRLMSKLASESETTRQFLLQNPNYSLVETLLE
AN0037.1  	TIQRTRKFEP---ADMSEPESPVMLSCYLRLIGHLSRQSAAVRDWMLHHSSSNIVSTLLT
          	     :  :       :****.::*.*****:.:*. :* ..*: :* . .  : ..:: 

MG08911.1 	LNSCHIPPRLRACGFYAIAALVRRKEQDDSNTMWLCIDAFVSGGFQAASNSRGLIKGQSH
NCU00273.1	LASAQIPGRLRACVFNAFRALMHRKTHQESEIMWDFIDLWLVGGWGPKADTQRTVAMLQP
FG05518.1 	LASSNIPPRLRGCTFMALRALMARKSLQEGALMWNCLDQWITGEYFLHPVTS--HRQSQQ
AN0037.1  	LCSGPLPSHLRATVFQALAGLMTDRSVENGNEMWLAIDQWISSGAMSASAHN-KVPLVSN
          	* *  :* :**.  * *: .*:  :  ::.  **  :* :: .     .         . 

MG08911.1 	SSAGVVDRVLDELSNGFEEPSAFINLINALVAPADQASLLRDALPFPENLGSSLRMPGIE
NCU00273.1	SPQAVMEAILDDISDGFEQPVAFIQLLISLIQPSIDDGELCDKLPFKETLGTSNRLSGVD
FG05518.1 	SPSVFMERIFDEISNGFEDPESFIQLLLSLISPAADSSPLNDGLPFPENLGSSSRMPGIE
AN0037.1  	PLIWHEQQAFEKIGESFDQANAFVVLILSLVAPAIDSAEYAIWVPFPESLGSSYRMPGIE
          	.     :  ::.:.:.*::. :*: *: :*: *: : .     :** *.**:* *:.*::

MG08911.1 	PYVDFVVGHVFSLKSKELQDVSQLRVLRVSCLEFILLCLNTFNEDLIILGNQTNVSVDLI
NCU00273.1	VYVDYVFGLVLTKKAMDITDPMQLRILQLSCLEFALASLASFNEDLIVLGNESNVNVDVA
FG05518.1 	IYVDFVIGLVFAQKANDLQDVNQTRVLRLSCLEFILICLNTFNEDLIIMANETNINVDSV
AN0037.1  	PYIDFIMGQALSRKVPDLNER-QSRLLTYNSLDFVLTCLRSFNETIVTALNETPATAEPN
          	 *:*::.* .:: *  :: :  * *:*  ..*:* * .* :*** ::   *::  ..:  

MG08911.1 	IATSDLATYVRLHPFARVMEWMFNGQVVDAIFETIHQQSSDIGSVSPDSPLILGIIRAVE
NCU00273.1	VSTSSLEAYVRLHPFARVMEWLFNEKVVNILVDIINRDTDVLGQSSHDSPVVLGVLRAVQ
FG05518.1 	IATTDLATYVRMHPFARVMEWMFNDKVMAALFNTIHQEPADVGNASPDSPLILGILRAVE
AN0037.1  	LKTSALLSYVRLHPFARVAEWLYNEDVIKTIFATANQEIADISRASPDSVLVSSLVKSIE
          	: *: * :***:****** **::* .*:  :.   :::   :.  * ** :: .::::::

MG08911.1 	VVSKVLELQDTYVDLVRQVIKQHTGQRHRHVPHASYASFEEGFAHHLEVVADLGRYCGLG
NCU00273.1	VMIKVLELQDTYLDLVRPLIKKQSGQRSRSVASGAYSAFEDGIMNHLSLVEDLGRFTNLG
FG05518.1 	VISKVLDLQATYLELVRPIIKLQSNQRRPPVANAAFASFEDGLVTRLNLVVDLGNYCGIG
AN0037.1  	VMCSIIDLQSTYLNIVRPFIKSQPGSRLN-VANSALSAFEDCILNNLQIIPKLCLYCGTG
          	*: .:::** **:::** .** :...*   *. .: ::**: :  .*.:: .*  : . *

MG08911.1 	HPELTMVCLKLLARISTSSKLISAWNTEPGRQSHRNKAVVALEKDGEADSISGSLIAELI
NCU00273.1	NPDLTWASLKLLEKISTSPEIISAWEP-DGRHAHRNKAIVQLERGGAGVSIAASLANTIA
FG05518.1 	HPELTLACLKLLEKMSSSSKITAVWSG-SSRQMHRNKAIVALEANGEHETISRSFVSELI
AN0037.1  	HQELTVSSLALLAKLSSSTRLNKLSTPELSRWRSSNKIVEVLSAENLDSLSLPFISQMEP
          	: :**  .* ** ::*:*..:      . .*    ** :  *.  .        :     

MG08911.1 	VPLDLAREADSPNYLIKTYILDFLYACLRASPDQPTIAHLLLGFKCGVSHLKVEPRGQFE
NCU00273.1	QTLDPVLEAESPDYQTKVFILDFLYKCLKANPDKPTIAHLLLGFRCEINGLAIEPNGAFD
FG05518.1 	TPLETGRETDSPAYVTKIYILDFLYQCLQETPRKPTIAHLLLGFKCGIDSLSVEAKGAFT
AN0037.1  	EVRELDAGPQASGYLIRHNLLELLNSSLTMIPDRPTVAHLLLGFGSVGNVLDVSSDGLFA
          	   :    .::. *  :  :*::*  .*   * :**:******* .  . * :.. * * 

MG08911.1 	ERTSLFHNLLRVLLETPFGDEELGMRTWLVALKRKVMGILQILWSSPLSSAIVLEE~LRS
NCU00273.1	IQRSLFHSLLNAFIG--------------------------------LSVYQEELE1LRA
FG05518.1 	SRTSLFHTILRLLLETPSGD-AQGMRQWLIAIKSRVMRILHILWSSPLSAPVVVEE~LRE
AN0037.1  	AGRSLLHAILGFVQTYP-DQLDDNIVSWMLHLKRTAFEVLKHLWSSKLSSYFTLAE~MRA
          	   **:* :*  .   . ..   .        .           :: **      * :* 

MG08911.1 	NDVLFHILLREESIQPDLMWDGVSMEERGFLLSDAAVGFSEFLATRQLTFDYLAIELCSV
NCU00273.1	TNFLFHTLLQEPQVQPHLRWDGVEAASPEFLVTDASHSYIEFLATRAMAFEYIAKELCSV
FG05518.1 	NEILFHLLLRETVINPDLPWEGENVALVQFPVTDGAIALIDFLSLRSMSLEYIAMELCSI
AN0037.1  	SGFLVSLFASQPLVGPHTLWDGLPIAAEQFWVCDSTDALAEFLLYRSYLFDYATTEIRSA
          	. .*.  :  :  : *.  *:*       * : *.: .  :**  *   ::* : *: * 

MG08911.1 	TQRRLPSLKRRIYDALNGQVTTANSDSPQSIPTIFDLYDFMPADGQWEIPLPEFTHFKGE
NCU00273.1	SQKRIPTVKRQIFDALLNGQIRADDEEPINIANIFDFFDFLNVD-FVDIPAPEFDIYRDI
FG05518.1 	AQNRMPSVKRRIFEALNGQIIGE-GDTPIPIPTIFDLYDFLLPEGVWEIPLPPLQYYKDL
AN0037.1  	AKMGSPTLLESILATLFG-NSTLDNGATIANPTVYDLFDFADLDVQFHAPPPNVEYLGGL
          	::   *:: . *  :* .     ..  .   ..::*::**   :   . * * .    . 

MG08911.1 	EFSMCLEGAHD-SVLMYNMERVKQVIALKRRQ--------DAQNGQIATAADLASVDREQ
NCU00273.1	DLTPCIEEDAM-TGSQFNVHKVHELALLKRAERLNRDNPEKKDSQALITQDELAAWDREQ
FG05518.1 	DLTACLENDTD-GNQIYNLERVKEVLLLKRGE--------AHDAGTLVAAQDLVAFEREE
AN0037.1  	NIDICEKPVEDGSLVLFNVAQVKELVKARREE---------LLSNGELRPQDDELFMAEA
          	::  * :    .    :*: :*:::   :* :                   :      * 

MG08911.1 	VILEEYLVYHNRQAAVSSERRKVLKAWVNLLMVMFEASDHQGSAKVAFLLQALQTILPTL
NCU00273.1	SLLLEYVIYRNRQKQFSAHRLKVLRAWTNILLVMFEANDFKGTPKIAFLLQALQAILPSL
FG05518.1 	SMIIEYLISSNRQKQLTTQSLKVLKTWTKLLLVMIESNDFKGSAQISFFLQALQSILPSL
AN0037.1  	DNLIEFLQARNQSRLINFNRHMALSSWAELLITILKCSELDRGRKSTFILHAIQLILPKL
          	  : *::   *:.  .. .   .* :*.::*:.:::..: .   : :*:*:*:* ***.*

MG08911.1 	ESFGSERPGEAFELAKLAKVLLFKLDIAAPDAEDKDAHTVGNLISEKLFQLFEVCLYAIG
NCU00273.1	EAFSTLSPAEAFELARVAKVLLFKLDLSDN-TDGATDKDIGNLISDKLFQLFQVCLTSIA
FG05518.1 	EAFASERPDEAFELAKLSKILLFKLDLSAS-ADDKQGSAIGNLISDKLFQLFQICLQAIG
AN0037.1  	ETAIEDNLPEALDLARLAESLVESLDATPAKLMPLR--RNGDLIDEKLHQLFQICLRGII
          	*:       **::**:::: *: .** :  .         *:**.:**.***::** .* 

MG08911.1 	RRTGSAELRSTYYAICYRYLIGIIDDGR---------DFVPGRRKAIKAVQVYGERLLTI
NCU00273.1	SWVGSADLRALYYSICYRYLTSVVDKTTDHTGTTTSNALATARSRAIRAIQAYGDRLLNV
FG05518.1 	KWAGTPELRSIYYSICYRYLTGMADQG----------PLTSNRPKTIKTIQVYGERLLNV
AN0037.1  	LSNGGVLLKETFYNICSIYVARIVSPDTG---------NATMRQHSQQVVKSSGRALIEA
          	   *   *:  :* **  *:  : .   .          .. * :: :.::  *  *:  

MG08911.1 	VCDDAFGGDATCQTSALILLGALTNLGRREDDG-----------QVVEALNRLNFIGVLV
NCU00273.1	ICDDAYGSDSTCQTAAMILLGALVHLDHTTTASGLQHTNDLDGSFVVTTLNRLNFIGVLV
FG05518.1 	ICDDAYGGEAQCQTAALILLNALISMGHQESEG-----------YVVETLNRLNFIGIMV
AN0037.1  	ICDDAYTGQETCRVSAVLLLNSLAALDKQADAT------------LAESIAQSNYLSLFL
          	:****: .:  *:.:*::**.:*  :.:                 :. :: : *::.:::

MG08911.1 	DSLKTILDDVTAARKA1G~NS--DQQE---------------------SYSHAKLALLLQ
NCU00273.1	DSLKTILKDWSAVTTS~P~TSSVEQQQQQQPPSSQQLTLATQSPATDSQYILSKLSLLLA
FG05518.1 	DSLRNIMQEWQEVFTS~G1TS--EQQ----------------------NYQNARLALLLE
AN0037.1  	DSIRVLPLELKNAPAN~G~RIPYIPTAN-------VANLSLTDTPLLLSYYQSLLSLLQQ
          	**:: :  :   .        .     .         . :  ...   .*  : *:**  

MG08911.1 	LCQTRDGAKYVLQANLLRAIEVSGLFAADPELQID1----------RSDTKALADHYDLL
NCU00273.1	LCRSKLGSKFVLQSNLFRALEVSGAFSTDPELFVR~QQQQNSSTGSTTYVRALERHYTLL
FG05518.1 	LAQTRSGAKYILHSNLFRALEISGLFGADPELQIN~----------STNPRALEQHYDLL
AN0037.1  	LCQTKPGATHVLKAGLFDAVRESHLFAADPDLGIA1T--------DIDNADALRKYYDLL
          	*.::: *:..:*::.*: *:. *  *.:**:* :           .     **  :* **

MG08911.1 	IRVMRVIGAAILS---RGAHNVPQGRRFLTEHRMLVMHVLKRSAGI--------------
NCU00273.1	VYLARIVSAAVMAGKKRGSHNALQGRKFLQGARGLVVQVLKMSVGIGGAGSGNLGVSMRA
FG05518.1 	AQVVRIVSAALVA---RGSHNVVQGRRFLTDHRMLVTHTLKRSAGIG-------------
AN0037.1  	DSVLRVIVTATFSRGLQNELMIEQTRAFLTENRQSMVGIFKRFAKIG-------------
          	  : *:: :* .:   :.     * * **   *  :   :*  . *.             

MG08911.1 	------------AAVDQKLEALVVGLADSFMVLITATEFLE0FEDTQGRGPQQQKKTGPV
NCU00273.1	VNGSAYSGADRDSQAQLELEERVDELAEAFMLLINATGFLE0FEE---EQTAAEKPKGDL
FG05518.1 	-----------SANADEGLDQKIEELAEGLMVIIAATRFLE0FEN-EGLPESTQQSHG--
AN0037.1  	------------GSAAPDHHDALTSIAKSYTALVAATNFLE0PRLTLRNAAYVIKRGIIF
          	            . .    .  :  :*..   :: ** ***  . :        :     

MG08911.1 	LFH------------------------------------------~--------------
NCU00273.1	LFH------------------------------------------~--------------
FG05518.1 	LFH------------------------------------------~--------------
AN0037.1  	ISDWDRTLRRSLNPSFFVQLPAPYSIKPPYIKTSQNWKLAAMVLV0MQQNSRSPPEQRSG
          	: . . :   : ..:   . .:. : ...  .::.. . ::      ...: :.... :.

MG08911.1 	------------------------------------------------------------
NCU00273.1	------------------------------------------------------------
FG05518.1 	------------------------------------------------------------
AN0037.1  	KRKATGKRKFPDQGELSQQPRHQQATISELLHRNQAQNQAQEQDDEHLPPTNKRVRPSSS
          	. .::.. . ..... :...  ..:: :.    ..:...:......  ..:..   .:::

MG08911.1 	------------------------------------------------------------
NCU00273.1	------------------------------------------------------------
FG05518.1 	------------------------------------------------------------
AN0037.1  	SIAANQPESGDMYSFSTAEPKPNGPNTGGLTLSNSTLQARPGTSSHQSNFTPHTGARRLV
          	: ::....:..  : :::........:.. : :.:: .: ..::: .:. :. :.:    

MG08911.1 	------------------------------------------------------------
NCU00273.1	------------------------------------------------------------
FG05518.1 	------------------------------------------------------------
AN0037.1  	VKNLRTGPRLNQDSYFDKVWGQLDAALSAIFSGGKPEISLEELYKGAENVCRQGRATILA
          	 ..  :..  ...:  ..  .. .:: ::  :..... : ..  ..:.. . .. ::  :

MG08911.1 	----------------------------------------------~-------------
NCU00273.1	----------------------------------------------~-------------
FG05518.1 	----------------------------------------------~-------------
AN0037.1  	KRLQERCRQHVTGKLHGSLVDKAQMAYNIETLRSVVEAWKEWQSML0ITVRWIFYYLDQS
          	.  .. . .  :..  .:  ..:. : . .:  :  .: .. .:    :        ..:

MG08911.1 	------------------------------------------------------------
NCU00273.1	------------------------------------------------------------
FG05518.1 	------------------------------------------------------------
AN0037.1  	FLLHSKEHPVIREMGLQQFRQHIYSDPTLQEKILQGACDLVSADRSDENGIVADSSLLRN
          	    :.. .   . . ..  .   :..: ...  ..:..  ::. :....  :.::   .

MG08911.1 	------------------------------------------------------------
NCU00273.1	------------------------------------------------------------
FG05518.1 	------------------------------------------------------------
AN0037.1  	AIELFHSLDVYVSGFEPVLVSGSKDFFSLWAQQEATGYLASYVENSHRLIEREMNRCEQF
          	: .   : .   :. ..   :.:..  :  :...::.  ::  ..:    . . . ... 

MG08911.1 	------------------------------------------------------------
NCU00273.1	------------------------------------------------------------
FG05518.1 	------------------------------------------------------------
AN0037.1  	SFNRTTKQKLSESLDQTLVTDQESVLLSQKDVLGLLRIGNKIALGQLYTLLERRDLGAKL
          	: . ::... :.: ..:  :...:   :...  .    ... : ..  :  .  . .:. 

MG08911.1 	------------------------------------------------------------
NCU00273.1	------------------------------------------------------------
FG05518.1 	------------------------------------------------------------
AN0037.1  	KGAFSTYIVEEGTGIVFDESEADMVVRLLSFKKQLDDIWNESFRRNEGLGHALRESFESF
          	..: ::   ...:.   ..:.:.      : ... ..  ..:   ... . :  .: .: 

MG08911.1 	------------------------------------------------------------
NCU00273.1	------------------------------------------------------------
FG05518.1 	------------------------------------------------------------
AN0037.1  	MNKGRKSDATGGTDNPKTGEMIAKYVDRLLRGGWRLAPTREAENMPLADEDAEINRQLDQ
          	 ... .:.::..:....:..  :.  .    ..   :.: .:.. . :...:. . . ..

MG08911.1 	-----------------------------------------------~------------
NCU00273.1	-----------------------------------------------~------------
FG05518.1 	-----------------------------------------------~------------
AN0037.1  	VLDLFRFVHGKAVFEAFYKNDLARRLLMGRSASDDAEKSMLARLKTE1CGSSFTHNLESM
          	  .      ..:  .:  ... :     . :::..:..:  :  .:. ..:: : . .: 

MG08911.1 	------------------------------------------------------------
NCU00273.1	------------------------------------------------------------
FG05518.1 	------------------------------------------------------------
AN0037.1  	FKDMEVARDEMSAYNSIKRERQTPLPVDLHVSVLSASAWPTYPDVQVRIPPEIATAISDF
          	 .. . : .. :: .: . . .:. . .   :  :::: .: .. .   ... ::: :. 

MG08911.1 	------------------------------------------------------------
NCU00273.1	------------------------------------------------------------
FG05518.1 	------------------------------------------------------------
AN0037.1  	EKFYDTKYNGRKLAWKHQLAHCQLRARFPNGNKELVVSSFQAIVLLLFNELPEGGTLNYR
          	..  .:. .. . : . . : ..  :  ......   :: .:      .. ....: .  

MG08911.1 	---------~--------------------------------------------------
NCU00273.1	---------~--------------------------------------------------
FG05518.1 	---------~--------------------------------------------------
AN0037.1  	QIQEATTLS1DQELTRTLQSLACAKYRVLSKKPKGRDVSPTDEFSYNASFTDPKFRIKIN
          	. ..::: : ... : : .: :.:.    :..... . :.:.. : .:: :...   . .

MG08911.1 	------------------------------------------------------------
NCU00273.1	------------------------------------------------------------
FG05518.1 	------------------------------------------------------------
AN0037.1  	QIQLKETKEENKTTHERVAADRHYETQAAIVRIMKSRKTITHAELVAEVIKATRSRGVLE
          	. . ..:.....:: .  ::.   .:.::     .: .: : :.  :.  .:: : .  .

MG08911.1 	----------~-------------------
NCU00273.1	----------~-------------------
FG05518.1 	----------~-------------------
AN0037.1  	PADIKKNIEK2LIEKDYMEREDGNRYQYVA
          	.:. ... ..   ...  . ....  .  :
```
